# Progress of Endogenous and Exogenous Nanoparticles for Cancer Therapy and Diagnostics

**DOI:** 10.3390/genes14020259

**Published:** 2023-01-19

**Authors:** Hideaki Fujita, Seiichi Ohta, Noriko Nakamura, Masaharu Somiya, Masanobu Horie

**Affiliations:** 1 Department of Stem Cell Biology, Research Institute for Radiation Biology and Medicine, Hiroshima University, 1-2-3 Minami-ku, Hiroshima 734-8553, Japan; 2 Institute of Engineering Innovation, The University of Tokyo, 7-3-1 Hongo, Bunkyo ku, Tokyo 113-8656, Japan; 3 Precursor Research for Embryonic Science (PRESTO), Japan Science and Technology Agency (JST), 4-1-8, Honcho, Kawaguchi-shi, Saitama 332-0012, Japan; 4 SANKEN (The Institute of Scientific and Industrial Research), Osaka University, 8-1 Mihogaoka, Osaka 567-0047, Japan; 5 Division of Biochemical Engineering, Radioisotope Research Center, Kyoto University, Yoshida-Konoe-Cho, Sakyo-ku, Kyoto 606-8507, Japan

**Keywords:** extracellular vesicle, DDS, imaging

## Abstract

The focus of this brief review is to describe the application of nanoparticles, including endogenous nanoparticles (e.g., extracellular vesicles, EVs, and virus capsids) and exogenous nanoparticles (e.g., organic and inorganic materials) in cancer therapy and diagnostics. In this review, we mainly focused on EVs, where a recent study demonstrated that EVs secreted from cancer cells are associated with malignant alterations in cancer. EVs are expected to be used for cancer diagnostics by analyzing their informative cargo. Exogenous nanoparticles are also used in cancer diagnostics as imaging probes because they can be easily functionalized. Nanoparticles are promising targets for drug delivery system (DDS) development and have recently been actively studied. In this review, we introduce nanoparticles as a powerful tool in the field of cancer therapy and diagnostics and discuss issues and future prospects.

## 1. Introduction

**Figure 1:**
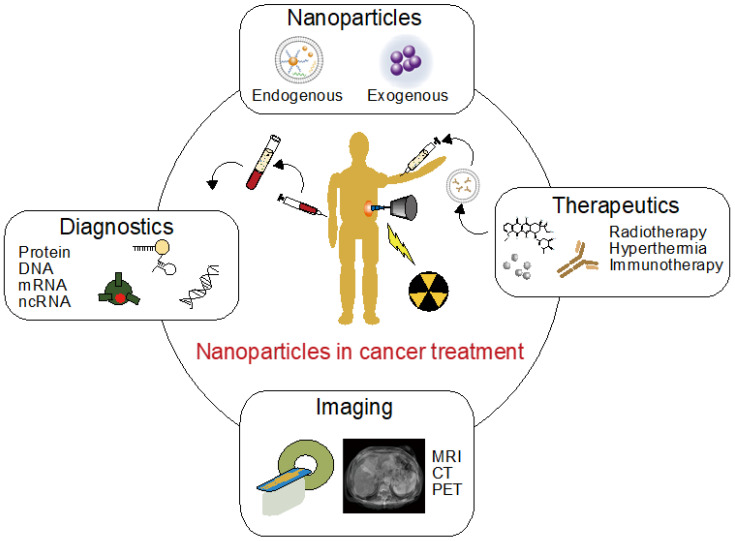
Schematic illustration showing the technologies introduced in this review. MRI, magnetic resonance imaging; CT, computed tomography; PET, positron emission tomography; DNA, deoxyribonucleic acid; mRNA, messenger ribonucleic acid; ncRNA, non-coding ribonucleic acid.

Cancer is a disorder that is hard to cure because it is basically a rebellion of self-cells, making it difficult to target only cancer cells in therapy. The basic approach against cancer is early detection, followed by chemotherapy and/or physical removal of the tumor if possible. Physical removal can be achieved by surgical operation or radiation therapy; both of these methods are highly invasive and require information about the tumor location. Chemotherapy is a less invasive therapy; however, it is only effective against certain types of tumors. In addition, chemotherapy has non-negligible risks of adverse effects [1,2]. Most anticancer drugs are designed to kill cancer cells that exhibit high proliferation behavior, and this characteristic causes adverse effects in healthy proliferative cells. Immunotherapy is another therapeutic method that has already been applied against cancer; however, like chemotherapy, it is only effective against certain types of cancer [3]. This may be attributed to the fact that cancer cell survival is related to immunosuppression and regulatory T cells protect cancer cells from the immune system. To achieve effective and minimally invasive cancer therapy, early diagnostics and high-accuracy imaging technologies are essential; treatment must be initiated as early as possible, and tumor location and temporal size changes should be monitored to analyze the therapeutic effect. However, current analysis technologies, such as fecal examination and projection radiography, cannot detect tumors until they reach a certain size. Thus, when a tumor is discovered, surgery is often an exclusive option. These hurdles hinder the development of minimally invasive cancer therapies. To overcome these hurdles, the use of nanoparticles is expected to be helpful. Figure 1 illustrates the technology introduced in this review. The history of nanoparticle research and development is very long, and the application range of nanoparticles covers not only the medical field, but also industrial fields and the space industry. In the field of cancer diagnostics and therapies, nanometer-sized particles called extracellular vesicles (EVs), such as exosomes, have attracted attention because they have been reported to be associated with malignant transformation of cancer [4,5]. Nanoparticles formed in vivo, such as EVs, are called “endogenous nanoparticles,” and their functions have been reported to be related to various diseases, homeostasis, and cancer. On the other hand, classical nanoparticles which are formed by chemical synthesis from organic and/or inorganic materials, are called “exogenous nanoparticles”. Highly functional exogenous nanoparticles have been reported to improve synthesis precision and analysis technologies, and these nanoparticles have been applied in various fields [6,7]. In this review, we mainly focused on EVs and summarize the research results in cancer diagnostics and therapies. A common issue in the application of endogenous and exogenous nanoparticles is the delivery of formed nanoparticles to the target location. For example, in terms of adverse effects, it is important to reduce the agent dose as much as possible while increasing drug delivery to the tumor. Research approaches using nanoparticles in these delivery technologies are also debated. Finally, we discuss future prospects and issues in cancer diagnostics and therapies using nanoparticles.

## 2. Extracellular Vesicles

### 2.1. Types of Extracellular Vesicles

**Figure 2:**
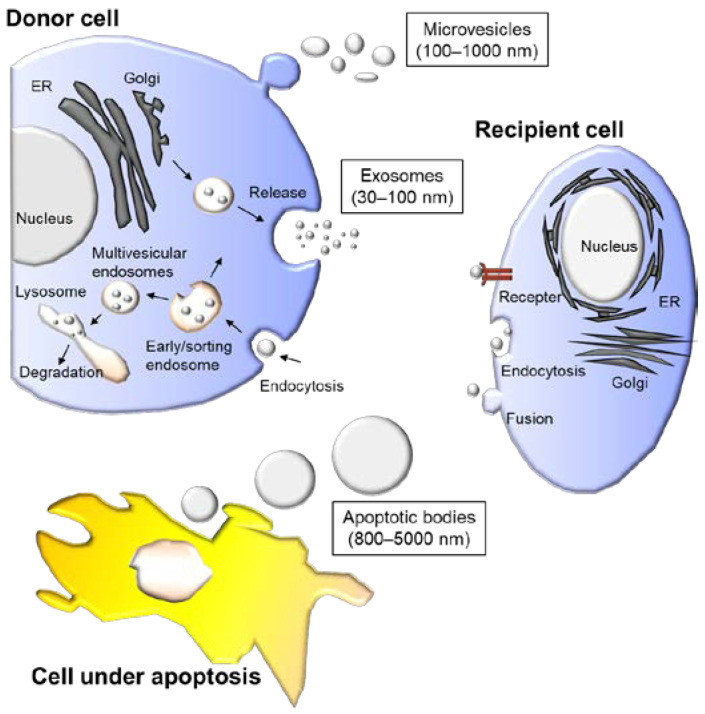
Schematic illustration of biogenesis of various extracellular vesicles (EV). EVs are categorized by their origin. Microvesicles are formed through budding whereas exosomes are generated from late endosomes. Apoptotic bodies are formed from apoptotic cells. Recipient cells may change their state through signaling from the EVs.

EVs, also known as microparticles or lipid vesicles, are secreted from cells with a lipid bilayer or multilayer structure. All living cells secrete EVs, which are generally categorized into several groups based on their origin, such as exosomes, microvesicles, and apoptotic bodies (Figure 2, Table 1) [8]. EVs carry various types of cargo, such as proteins, deoxyribonucleic acid (DNA), ribonucleic acid (RNA), lipids, and metabolites [9], and they are decorated by surface molecules, which are crucial for the targeting of recipient cells. EVs may be important for intercellular communication and can modify the state of recipient cells with their cargo or surface molecules. Because various types of EVs are secreted from the same cell, they are heterogeneous in size, composition, and origin [10,11]. Exosomes are the most studied EVs and originate from endosomes; early endosomes develop into late endosomes and form multivesicular bodies containing numerous luminal vesicles. Microvesicles are secreted from cells through budding of the plasma membrane. Apoptotic bodies with diameters ranging from 50 nm to a few micrometers are generated through the disassembly of cells during apoptosis. These EVs are currently gaining attention in various fields of science owing to their growing significance in diagnosis and therapy. The liquid biopsy of EVs in body fluids is an emerging diagnostic tool. Apart from circulating tumor cells in the bloodstream, EVs are also targets for liquid biopsy [12]. Because EVs are present in all body fluids and carry nucleic acids, a minimally invasive diagnosis is possible. Monitoring tumor progression or deciding on optimal care is possible by investigating DNA errors derived from tumor cells in EVs. In addition to tumor-derived DNA, surface or luminal proteins or microRNA (miRNA) cargo in EVs may reflect tumor progression and thus may be useful biomarkers for liquid biopsy. Exosomes and microvesicles can be targets for liquid biopsy, and although their origin is different, their composition is similar. EVs secreted by cancer cells are thought to play a role in tumor formation, transformation, and metastasis. Recent advances in liquid biopsy are discussed in detail in the following sections.

### 2.2. Therapeutic Use of EVs

The therapeutic use of EVs has been proposed because they can modify the state of target cells. In particular, EVs derived from immune cells can prime early T cells, differentiate mature T cells, and develop effector functions, such as antigen presentation and activation of immune cells [13]. The EVs’ functions on immunity have been focused by these discoveries, and immunotherapy using these functions is expected. Wolfers et al. showed that EVs derived from cancer cells possess cancer antigens, and these EVs could induce cancer antigen-specific immune response and anticancer efficacy [14]. This research raised expectations to achieve cancer vaccine therapy using antigen presentation [15]. Vaccine therapy using cancer cell derived EVs has an advantage unlike conventional immunotherapy, it does not require the identification of cancer antigens. On the other hand, the vaccine therapy using cancer derived EVs has shown anticancer efficacy, however, the therapeutic effect is still insufficient. Therefore, many researchers strive to elucidate the mechanism of EVs on human immunity [13]. Immunotherapy using EVs with anti-inflammatory properties is another type of immunotherapy that differs from vaccine therapy. In addition, EVs derived from cancer cells possess cancer antigens and can therefore be used for cancer immunotherapy by providing cancer-specific antigens to the immune system [14]. EVs derived from mesenchymal stem cells (MSC) are attractive for therapeutic use because of their low immunogenicity and ability to enhance injury recovery [16,17]. MSC-derived EVs have been reported to possess antioxidant, anti-inflammatory, and anti-apoptotic properties [18,19]. Furthermore, recent studies have shown that they protect cardiomyocytes from ischemia. Thus, EVs can function as drugs.

In addition to using EVs as drugs, EVs may deliver cargo into target cells, and the use of EVs as drug delivery system (DDS) carriers is gaining interest in the medical field. When using EVs as therapeutic DDS carriers, it is necessary to load the drug to be delivered into the vesicles [20,21]. This can be achieved by preloading the therapeutic nucleic acid into the cells producing the vesicles or introducing the drug into purified EVs. In the former case, although the mechanism by which nucleic acids and other inclusions are incorporated into the exosomes has not been fully elucidated, various methods have been developed [22,23]. In the latter case, where cargo is encapsulated into purified EVs, there is still a significant barrier to achieving efficient drug encapsulation [24]. Hydrophobic drugs, such as anticancer drugs, can be loaded passively through hydrophobic interactions with lipid bilayers [25]. However, owing to the hydrophobic lipid bilayer, hydrophilic drugs such as nucleic acids require a technique to permeate the membrane. Electroporation and sonication have been used to create pores in the EV membranes [26,27]. However, it has been highlighted that excessive physical stimulation may induce the aggregation of EVs, thereby altering their morphological characteristics, and changes in the surface potential of the membrane may increase cytotoxicity. Various attempts were made to increase drug-loading efficacy such as use of mesoporous material, use of acoustofluidic device, or dimerization of drugs, [28,29,30].

For the delivery of EV cargo into target cells, especially when the drug of interest is encapsulated inside EVs and is unable to penetrate the lipid bilayer by itself, the drug must be released from the inside of EVs to exert its biological function after cell entry. Since EVs are mostly internalized by endocytosis, membrane fusion between the endocytic membrane and the EV membrane must take place. Whether EVs exhibit membrane fusion activity is a subject of considerable debate [31,32,33,34]. Several reports have demonstrated that EV-mediated cargo delivery is an inefficient process where less than 0.2% of recipient cells functionally receive the RNA cargo from EVs in vitro [35], while engineering EVs with virus-derived fusogenic proteins significantly enhances cargo delivery [36,37]. These studies strongly suggest that engineering EVs to enhance cytoplasmic delivery is the most critical issue for the DDS application of EVs. Because exogenous nanoparticles can be a promising DDS carrier for cancer therapeutics, various nanoparticles loaded with anti-cancer drugs have been developed. Drugs such as paclitaxel, doxorubicin, or vincristine were loaded into liposomes and used as anti-cancer drugs at clinics [38,39]. Other than anti-cancer drugs, reagents which generate heat by external energy can be incorporated into nanoparticles and used for hyperthermia therapy. Magnetite is a colloid of Fe_3_O_4_ iron oxide that can be used as a contrast agent for MRI; magnetite generates heat when stimulated with alternating magnetic fields [40]. Agents for photothermal therapy such as metal nanoparticles or polymers can also be delivered to tumor sites by nanoparticles [41]. Various nanoparticles have been in use since the 1990s, such as liposomal daunorubicin or doxorubicin [42], since efforts were made to widen the variety of drugs or increase the efficacy of the medicine [43]. The global market of nanomedicine is rapidly growing with an estimated business of USD 293.1 billion in 2022 [44,45].

### 2.3. Biofabrication of EVs

As all cells secrete EVs, cultured cells are the best candidates for fabricating EVs for therapeutic use. In general, the medium used for mammalian cell culture contains serum, including animal derived EVs. Thus, the use of a defined medium without a serum component is indispensable [46]. The source cells of EVs are chosen for their therapeutic use but can even be fabricated from the same cell type. EVs have heterogeneous physical properties such as size, density, and shape, as well as composition of cargo and surface molecules [11,47,48]. These heterogeneities may affect the efficacy of EVs when applied as therapeutic drugs, and the properties of carriers when applied as DDS carriers. EVs can be isolated using various methods, such as differential centrifugation, density gradient centrifugation, ultrafiltration, affinity chromatography, precipitation, and size exclusion chromatography [49,50]. The complete separation of EVs from other biosubstances of similar size, such as lipoproteins and protein aggregates, is currently difficult. Various EV separation kits are available which have been reported to have higher separation efficiency compared with conventional ultracentrifugation methods [51,52]. Size exclusion chromatography methods have also been used to separate exosomes from protein aggregates, which reportedly have less contamination than other methods [53]. The use of a microfluidic device or tangential-flow filtration may reduce contamination.

Both biochemical and physical properties are used to evaluate the quality of EVs. Biochemical analysis uses proteomic, genomic, and lipidomic approaches [54]. Western blot analysis is a common biochemical approach for bulk EV samples, by which EV-related proteins, such as tetraspanins (CD9 and CD63), have been confirmed [55]. The morphological characteristics of EVs can be evaluated using electron or atomic force microscopy [56,57]. Optical imaging is not possible because EVs are often smaller than the wavelength of visible light. However, the use of a fluorescent microscope to labeled EVs enables visualization of their presence [58,59]. The size and surface charge of EVs can be estimated using methods such as dynamic light scattering or nanoparticle tracking analysis, and zeta potential analysis, respectively [60,61,62]. None of the single methods can quantify EVs’ characteristics. Therefore, a combination of approaches is required.

## 3. EV-Targeted Diagnostics Using Synthetic and Functional Nanoparticles

### 3.1. EV-Based Liquid Biopsy for Cancer Diagnostics

As mentioned in the previous section, EVs also play important roles in intercellular communication involved in tumor development and are expected to be a promising source of biomarkers for biofluid (e.g., blood and urine)-based cancer diagnostics, called liquid biopsy [63]. For instance, surface proteins of exosomes represent their origin and alteration of the parent cells and are therefore expected to be cancer biomarkers. Examples include prostate cancer antigen 3 [64] and survivin for pancreatic cancer [65], CD24 for breast cancer [66] and ovarian cancer [67], and CD9 and CD147 for colorectal cancer [68]. Nucleic acids contained in exosomes, such as miRNAs, messenger RNA (mRNAs), and long noncoding RNAs, are also promising biomarkers for cancer diagnostics [69,70]. Among these, exosomal miRNAs have been intensively investigated because of their relatively high stability against enzymatic degradation [71]. Exosomal miR-23b-3p, miR-10b-5p, and miR-21–5p have been reported as prognostic biomarkers for non-small cell lung cancer [72], whereas miR-141 has been reported as a biomarker for prostate cancer [73]. Various other examples can be found in previous reviews [74].

**Figure 3:**
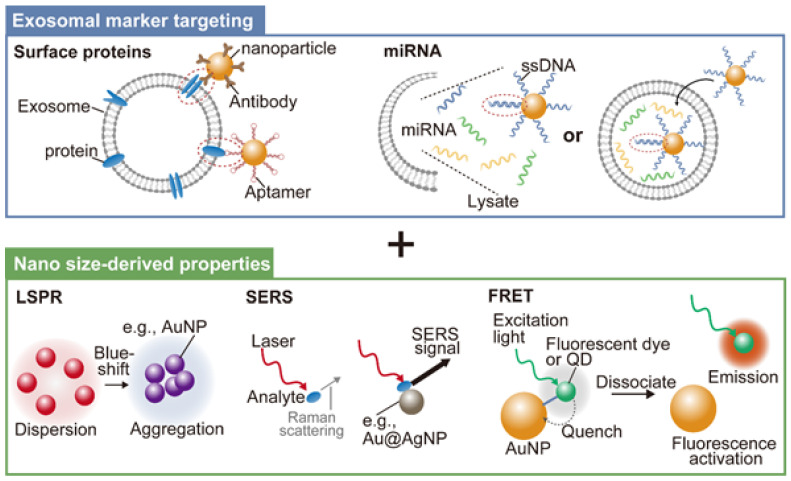
Synthetic nanoparticle-based detection of exosomal surface proteins and miRNAs. Synthetic nanoparticles can target specific exosomal markers via surface molecules, such as antibody, aptamer, and single stranded DNA (ssDNA). They also exhibit size-derived specific properties including localized surface plasmon resonance (LSPR), surface enhanced Raman scattering (SERS), and Förster resonance energy transfer (FRET). By integrating these targeting properties and size-derived functions, synthetic nanoparticles can be a promising tool for the detection of exosomal surface proteins and miRNAs.

Due to the heterogeneity of samples, as well as the small quantity of target analytes, sensitive and selective analysis of EVs in a facile and inexpensive way has been a challenge in the field of diagnostics. In addition to widely used methods, such as enzyme-linked immunosorbent assay for protein markers as well as quantitative real-time polymerase chain reaction (qRT-PCR) and next-generation sequencing for nucleic acid markers, highly sensitive and selective EV analysis methods using nanoparticles have recently been investigated [75,76]. Nanoparticles are known to exhibit specific functions derived from their nanometer size, which is an intermediate between atomic and bulk scales. For instance, semiconductor nanoparticles, which are often called quantum dots (QDs), exhibit fluorescence derived from the quantum-confinement effect, whereas metal nanoparticles show strong optical absorbance at specific wavelengths owing to localized surface plasmon resonance (LSPR). The following sections introduce the use of these synthetic, functional nanoparticles for EV analysis (Figure 3).

### 3.2. Use of Synthetic Nanoparticles for Analyzing Exosomal Surface Proteins

Antibody surface modification is the main strategy for targeting synthetic nanoparticles to cancer-related exosomal surface proteins. Aptamers, which are short sequences of artificial DNA or RNA that bind a specific target molecule, have been another choice owing to their smaller size and lower cost compared with those of antibodies. Binding of synthetic, functional nanoparticles to target exosomal surface proteins through these targeting moieties enables their effective capture and sensitive detection via nano size-derived functions.

For example, LSPR-based detection of exosomal surface proteins utilizes the strong optical absorbance of plasmonic nanoparticles at a specific wavelength around the red color, which can be blue-shifted by their aggregation. Jiang et al. non-covalently coated gold nanoparticles (AuNPs) with a panel of aptamers that can specifically bind to exosomal surface proteins, i.e., CD63, EpCAM, PDGF, PSMA, and PTK7 [77]. After mixing the aptamer-coated AuNPs with target exosomes in a high-salt concentration solution, binding of the aptamers to target exosomal surface proteins induces the aptamer displacement from the AuNP surface, leading to the aggregation of AuNPs and a shift in LSPR-derived absorbance. By constructing an aptamer-coated AuNP-based detection panel for the above five cancer protein markers, the authors demonstrated surface molecular profiling of exosomes isolated from various cancer cell lines.

When metal nanoparticles bind to molecules, Raman scattering from the molecules is significantly improved by up to 10^10^–10^11^ times, enabling their sensitive detection at the single-molecule level. This phenomenon is called surface enhanced Raman scattering (SERS) and has also been used for exosomal surface protein detection. Li et al. developed Au-core/Ag-shell nanoparticles (Au@Ag NPs) modified with antibodies for the exosomal cancer protein marker, migration inhibitory factor, as SERS tags [78]. Exosomes were first captured by antibody-immobilized substrate and then labelled with Au@Ag NPs, resulting in the specific detection of pancreatic cancer-derived exosomes via SERS with a detection limit of ca. 9.0 × 10^−^^19^ M. The developed SERS assay enabled the classification of pancreatic cancer patients and healthy individuals, metastasized tumors and metastasis-free tumors, and tumor node metastasis P1–2 stages and P3 stage.

In addition to LSPR and SERS, other nano size-derived functions of synthetic nanoparticles have also been utilized for the analysis of exosomal surface proteins [79,80,81,82]. For example, the magnetic property of superparamagnetic iron oxide nanoparticles has been used for the capture and isolation of exosomes with specific target surface proteins [82], whereas the peroxidase-mimetic activity of iron oxide nanoparticles has been used for the detection and analysis of exosomal surface proteins [83].

### 3.3. Use of Synthetic Nanoparticles for Analyzing Exosomal miRNAs

While exosomal protein markers are mainly expressed on the cell surface, exosomal nucleic acid markers, including miRNAs, are encapsulated inside exosomes. Analysis of exosomal miRNAs usually requires RNA extraction, followed by amplification and detection of target miRNAs using the qRT-PCR method [84]. Recent studies have also examined the direct detection of miRNAs in a single exosome, which requires membrane fusion or membrane penetration of the detection probes together with their detection using a single vesicle imaging system [85,86]. To target these exosomal miRNAs using synthetic nanoparticles, the main strategy is to modify their surfaces with single-stranded DNAs (ssDNAs) which have a complementary sequence to the target miRNAs. The ssDNA-modified nanoparticle surface can bind to target miRNAs via double helix formation, resulting in sequence-specific targeting.

AuNPs exhibit strong quenching against a wide range of fluorophores via Förster resonance energy transfer (FRET) when they are close to the particle surface. Together with their large surface area to load signal amplification agents, AuNPs have been used as a platform for fluorescent detection of biomolecules, including exosomal miRNAs [87]. Zhai et al. reported an Au nanoflare-based fluorescent probe for detection of the exosomal breast cancer miRNA marker, miR-1246 [88]. They prepared ssDNA-modified AuNPs, followed by hybridization with Cy3-modified ssDNA, which was partially complementary to the ssDNA on the AuNP surface. At this initial state, because Cy3 is close to the AuNP surface, fluorescence from Cy3 was quenched by FRET. After application to the plasma sample, the Au nanoflare probe penetrates the exosomal membrane to target the internal miRNAs. Because the ssDNA on the AuNP surface was designed to be complementary to miR-1246, exosomal miR-1246s could bind to the ssDNA on the AuNP surface via toehold-mediated strand displacement, resulting in the release of Cy3-mediated ssDNA from the AuNP surface, leading to the activation of the fluorescent signal. By measuring plasma miR-1246 levels using the aforementioned probe, successful identification of breast cancer patients from healthy controls was demonstrated.

In addition to direct labelling, amplification of fluorescent signals is a promising strategy for sensitive miRNA detection. Degliangeli et al. reported an AuNP-based fluorescent amplification and detection platform for cancer-related miRNA markers, miR-21 and miR-203 [89]. AuNPs were coated with fluorescein mercuric acetate (FMA)-modified ssDNA, which could bind to target miRNAs via duplex formation. On adding ssDNA-modified AuNPs to samples with duplex-specific nuclease (DSN), target miRNAs could bind to ssDNA on the AuNP surface via duplex formation. Subsequently, activation of the FMA fluorescent signal was induced by DSN-mediated degradation of the formed duplex. As the target miRNAs are released after DSN-mediated duplex cleavage, they can be reused for further fluorescent activation cycles, resulting in the amplified fluorescent detection of miR-21 and miR-203. A similar amplification strategy with an enzyme-free catalytic DNA reaction, in which QDs were used as a fluorescent agent instead of small molecular fluorophores, was also reported [90].

In addition to the above strategies, SERS and electric signals have been used for synthetic nanoparticle-based miRNA detection [91,92]. Further challenges in this field include the development of a detection platform for multiple miRNA panels. Recently, cancer diagnostics using several tens of miRNAs (typically around 20 types) as a panel has been recognized as a promising approach [93,94]. The development of novel synthetic nanoparticle-based platforms that can achieve facile, sensitive, and multiple detections of exosomal miRNA markers is expected to further accelerate the future clinical translation of this miRNA panel-based diagnostic approach.

## 4. Exogenous Nanoparticle-Based In Vivo Diagnostic Imaging

As mentioned above, EVs are prospective targets for cancer therapies and diagnostics. While the liquid biopsy-based analysis of EVs provides information for the origin and characteristics of cancer, the size and location of tumor are also important information for cancer therapy and diagnostics. In particular, for non-invasive cancer therapy methods, such as anticancer drugs and radiation, this monitoring information is important for evaluating the treatment effect. This information is also essential for the efficiency and safety of surgical procedures in the surgical therapeutic field. For achieving the accurate monitoring of tumor size and location, exogenous, synthetic nanoparticles can play important roles. In this section, we introduce synthetic nanoparticle-based in vivo imaging technologies for cancer therapy and diagnostics.

To visualize formed tumors, molecular imaging has attracted much attention as a powerful technology for understanding cancer biological phenomena and medical applications as non-invasive diagnostic techniques. In particular, nanoparticulated imaging agents have been explored as versatile probes for molecular imaging because they have characteristic functions derived from their size and can be modified with ligands that are well-suited to target specific biomolecules. Numerous functionalized nanoparticles have been developed and proposed as imaging agents for various imaging modalities such as magnetic resonance imaging (MRI), X-ray computed tomography (CT), positron emission tomography (PET), and fluorescent imaging. Regardless of the modality, nanoparticulated imaging agents have demonstrated outstanding performance enabled by fine-tuning the nanoparticle size, surface properties, composition, and other characteristics.

MRI, CT, and PET imaging technologies have been widely used as powerful tools for noninvasive diagnostic imaging because of their excellent penetration depths. Paramagnetic gadolinium (Gd) complexes have been commonly explored as MRI contrast agents, mainly for vascular visualization and brain tumor detection. Nanoparticulated Gd contrast agents improve the circulation time and target specificity; however, the relatively low relaxivity and high dose requirement are potential issues. Superparamagnetic iron oxide nanoparticles are expected to improve contrast efficacy compared to conventional Gd-based nanoparticles, which can be further tuned by adjusting the size and composition of the nanoparticles [95]. Their use can also help avoid potential side effects caused by the use of ionized Gd and the high requisite doses of Gd-based contrast agents. Recently, iron oxide-based superparamagnetic nanoparticles such as “Ferridex” and “Resovist**”** have been approved by the Food and Drug Administration in the United States for liver cancer detection; however, a higher spatial resolution is required for a more accurate diagnosis. Furthermore, PET has emerged as a clinical imaging modality because of its advantages, including excellent penetration depth and quantitative capability. PET imaging via radiolabeled nanoparticles has improved PET contrast efficiency and accelerated the quantitative evaluation of drug delivery to tumors because of the reduced risk of radioisotope detachment [96].

Fluorescence imaging is also a powerful modality for molecular imaging because of its high spatial and temporal resolutions. Although many techniques for fluorescent imaging have been proposed as advanced modalities of molecular imaging with higher spatial resolution, these technologies face limitations in the detection limit due to restrained brightness and the inevitable photobleaching of small fluorescent molecules. To overcome these limitations, many studies have focused on developing nanoparticle-based fluorescent probes. Quantum dots (QDs) consisting of semiconductor nanocrystals have been reported as effective imaging probes for visualizing cellular membrane proteins and intracellular components [97]. Although the brightness and long-term stability make QDs candidates for further applications, such as 3D confocal imaging and in vivo targeted real-time imaging, cytotoxicity due to the inherently toxic heavy metals in its core (e.g., cadmium and selenium) has been a controversial issue. Therefore, QDs composed of less toxic semiconducting nanocrystals (e.g., indium phosphide and silicon) have attracted considerable attention as bright and biocompatible probes for fluorescence bioimaging [98]. In addition to low-toxicity QDs, polymer dots based on conjugated semiconducting polymers have been investigated as alternative fluorescent probes without considering the cytotoxicity caused by ionized heavy metals [99].

These imaging modalities have various advantages and disadvantages. For instance, MRI, CT, and PET have high penetration depths, however, their spatial resolution is limited to the millimeter scale. In contrast, fluorescent imaging has high spatial resolution at the subcellular scale, however, its penetration depth is limited to a few centimeters. To benefit from each advantage, multimodal imaging has generated considerable research interest in increasing the accuracy of diagnosis using complementary information from different imaging modalities [100]. The fabrication of nanoparticulated imaging probes allows them to exhibit multifunctional characteristics; therefore, the strength of each imaging modality can be integrated into a multimodal nanoparticle. For instance, ^64^Cu-labelled and RGD (Arg-Gly-Asp) peptide-conjugated iron oxide nanoparticles were developed as PET/MR dual-modality imaging probes for tumor integrin expression, whereas the near-infrared fluorescent (NIRF) dye ZW800 loaded with silica nanoparticles labelled with Gd ions and ^64^Cu was developed as PET/MR/optical imaging probe for tumor-draining sentinel lymph nodes [100]. In addition to diagnostic imaging, these multifunctional nanoparticles can also serve to monitor therapeutic efficacy of cancer treatment (theranostics) [101]. For example, croconaine dye-based nanoparticles were developed for photoacoustic/fluorescent imaging-guided photothermal therapy [102], while SPIO and olaparib-loaded exosome extracted from hypoxic cells was investigated for magnetic particle imaging and therapy of hypoxic region in tumor [103]. Furthermore, multimodal nanoparticles have also improved the accuracy and quality of in vivo cellular tracking, leading to a great contribution to animal cell-based diagnostics and therapy. For instance, Huang et al. developed a mesenchymal stem cell (MSC)-based multifunctional theranostic platform for targeted delivery of MSCs to glioblastoma and multimodal imaging with hyaluronic acid-coated mesoporous silica nanoparticles with green fluorescent dye (FITC), NIRF dye (ZW800), Gd^3+^, and ^64^Cu [104]. In vivo multimodal imaging with optical and magnetic resonance imaging and PET successfully revealed the feasibility of tumor tropism-facilitated delivery of their multifunctional MSC platform with improved tumor accumulation. Nanoparticle-based multimodal imaging improves the properties of MSC-based theranostics and immune cell-based theranostics for cancer. A dual-modal PET/NIRF nanoparticle-based imaging probe for labelling chimeric antigen receptor (CAR) T cells achieved long-term whole-body immune cell tracking in a mouse model of carcinomatosis [105]. This type of nanoparticle-based multimodal cellular tracking technique is crucial for advancing cell-based therapy to investigate the fate of administered cells and the therapeutic effect. These nanoparticle-based multimodal cell-tracking systems are expected to lead to the next generation of theranostics for future clinical applications. The core of QDs often contain heavy metals such as selenium, cadmium, or lead, which are toxic to the human body. To reduce toxicity, these metal cores are covered with non-toxic polymers, however, contamination during manufacturing process or leakage from deficient QDs cannot be ignored. Gd used for MRI is also toxic and bioaccumulative, thus, must be used with a chelation compound to reduce toxicity and ensure rapid elimination from the body.

## 5. Nanoparticle-Based Biological Drug Delivery System (DDS)

As previously mentioned, various nanoparticles have been developed and applied in cancer therapy, diagnostics, and imaging. As therapeutic methods using biomolecules (e.g., artificial recombinant protein, nucleic acid) are expected in the field of cancer therapy, developing innovative technology to deliver these materials to a specific place in the body (DDS technology) is essential. DDS is a key technology for improving the therapeutic effect of drugs by optimizing the physicochemical properties of active pharmaceutical ingredients (APIs), improving pharmacokinetics, and targeting specific cells [106]. Compared with conventional small-molecule drugs, the bioavailability and efficacy of biopharmaceuticals, such as recombinant proteins manufactured by animal cells, inherently rely on DDS technology for several reasons. First, the molecular size of therapeutic biomolecules is considerably larger than that of small molecules; hence, biomolecules rarely penetrate the cell membrane and reach inside the cells where the drugs function. Thus, the intracellular delivery of biopharmaceuticals is essential for achieving the therapeutic effect. Second, some biopharmaceuticals are prone to enzymatic degradation in the body. For example, therapeutic nucleic acids, especially RNA, are easily degraded by the abundant nucleases in the body. These biomolecule-derived APIs should be protected by extensive chemical modifications or delivery vesicles [107]. Therefore, DDS technology is highly desirable for material protection and targeting; hence, optimization of DDS is a pivotal step in the development of biopharmaceuticals.

DDS is mainly achieved using two methodologies: chemical and biological approaches. Chemical approaches involve the use of chemical substances or synthetic nanoparticles to deliver drugs. Pharmacokinetics can be improved via chemical conjugation of the targeting moiety to the APIs or attachment of the polymers (e.g., polyethylene glycol) to APIs. Synthetic nanoparticles, such as liposomes, inorganic materials, and polymer materials, have been used to encapsulate APIs for delivery. In contrast, biological approaches use biomolecules or biological vectors to achieve efficient delivery of APIs. Biomolecules or biological vectors are nanometer-sized particles, similar to other synthetic nanoparticles. Nanoparticles, such as viral vectors, are powerful tools for delivering genetic information of therapeutic molecules in the form of DNA or RNA. Currently, adeno-associated virus (AAV) vectors are used for gene therapy, adenovirus vectors are used as prophylactic vaccines against infectious diseases, and lentivirus or retrovirus vectors are used for ex vivo gene therapy, such as CAR-T cell therapy. Virus-like particles (VLPs) mimic the delivery mechanism of viral vectors; moreover, they avoid genetic materials; thus, they are safer than viral vectors and are not involved in gene insertion into target cells [108]. More recently, EVs secreted by all cell types have been widely studied as novel biological vectors for DDS [109].

It is well known that biotechnology and bioengineering are central disciplines when developing a biological DDS. Using technical knowledge from these disciplines, the above biological DDS could improve existing biologics and develop novel therapeutic modalities. Engineering of existing viral vectors is an emerging topic, and several studies have shown that in vivo tropism and functionality of viral vectors can be designed. Ogden et al. analyzed the fitness of the AAV vector using a mutant capsid library and found that certain mutations in the capsid protein affected the biodistribution of recombinant AAV vectors. This machine-guided design is a powerful tool for the identification of mutant AAV vectors with favorable properties for in vivo gene therapy [110]. Mihara et al. demonstrated that a targeting moiety, a macrocyclic peptide, can be inserted into the surface-exposed loop of the AAV capsid protein, changing the tropism of the AAV vector [111]. The in silico design of proteins may also be an attractive strategy for developing a new protein-based DDS [112]. These technological advancements in biological nanoparticle-based DDS have evolved with the changing cancer therapy approach.

In addition to the importance of functional modifications, mass-scale production is a critical issue in the development of biological DDSs. Although the production process of biologics has already been established, the production and purification of biological nanoparticles, such as viral vectors and EVs, remain challenging because of the complexity and difficulties of the purification and scale-up processes [113]. Generally, the production yield of biological nanoparticles is substantially lower than that of conventional biologics (e.g., therapeutic immunoglobulins) because of their inherent properties and the lack of efficient production technologies. Establishing a balance between the yield and purity of the final product is a trade-off when producing biologics. Therefore, the development and optimization of an efficient production process is key for reducing the manufacturing costs of biological nanoparticles and achieving affordable therapeutics.

Nanoparticle-based biological DDS, such as biomolecules and biological vectors, are a promising platform for the delivery of cancer therapeutics. Owing to recent fundamental discoveries in biology, such as gene-editing technologies, biological DDS could deliver a novel type of therapeutics to the target site in the body. Therefore, biotechnology-based biological DDS is the foundation for next-generation cancer therapeutics.

## 6. Future Prospect

This brief review summarizes the current approaches to cancer therapy and diagnostics using biological nanoparticles generated by cells and other organisms (endogenous nanoparticles) and artificial nanoparticles generated by chemical synthesis and other methods (exogenous nanoparticles). For endogenous nanoparticles, we introduced EVs and DDS technology using virus vectors, which have attracted particular attention in recent years for cancer therapy and diagnostics.

Recently, gene therapy for cancer treatment has been highly anticipated with the improvement of genome editing technologies such as CRISPR/Cas9. The treatment strategy using genome editing technologies is to attack target cancer cells by returning genome-edited T cells to the body. This genome editing is performed in vitro. On the other hand, RNA interference technology has also been expected for cancer treatment. This technology specifically suppresses gene expression with a short double-stranded RNA (siRNA) by degrading its sequence-specific target mRNA [114]. To enhance the treatment effect, siRNA should be delivered to targeted cancer cells because the inhibitory effect of RNA interference on gene expression is restricted to cells where siRNA is present. The expectation of gene therapy peaked in the 2000s; however, this expectation collapsed with unfortunate accidents, which might be attributed to DDS [115,116,117]. To avoid these accidents, DDS need to be improved in terms of their function and general understanding of endogenous nanoparticle safety. There are many unclear points regarding endogenous microparticles, including their intracellular formation process, extracellular dynamics, and even their biological significance, which is an essential issue in the development of cancer diagnostic and therapeutic techniques for these particles. Adverse effects, such as allergic reactions, have been reported in clinical trials with EVs, showing the need for further investigation [118]. Furthermore, because of the high manufacturing cost in exchange for safety and high medicinal effects, endogenous nanoparticles have a bottleneck for clinical application. On the other hand, manufacturing cost of exogenous nanoparticles is low and it is easy to add various functions. We introduced synthetic exogenous nanoparticles for imaging. In addition to conventional methods using gene-editing technologies and protein engineering, the modification and functionalization of endogenous nanoparticles using scientific manufacturing technologies of exogenous nanoparticles have recently been developed [119,120]. Moreover, research articles relating to endogenous–exogeneous hybrid nanoparticles constructed in complexes with nanocarriers or inorganic particles have significantly increased [121,122]. As mentioned in this review, nanoparticles have great potential as medical materials to eradicate cancer. On the other hand, despite the intensive research and success in treating tumors in mouse models, only a few non-targeted nanoparticle formulations, such as Abraxane and Doxil, have been clinically approved at present [123]. A recent review suggested that the average delivery efficiency of previously reported nanoparticles into solid tumor was 0.7% and did not change much over past 10 years [124]. It has also been reported that since the diffusion of nanoparticles in tumor is strictly prohibited by the dense ECM network, most nanoparticles extravasated from blood vessels cannot reach the tumor core and stay near the blood vessel wall [125]. To overcome these issues for actual clinical translation, revisiting the tumor delivery strategy of nanoparticles based on the deep understanding on their interaction with biological environment is required. In addition, applying the DDS technology to diseases other than tumor has also attracted much attention: examples include antibody delivery into brain for neuro-degenerative diseases [126] or antisense oligonucleotide delivery for RNA interference for muscular dystrophy [127]. Through these investigations, future research in cancer therapy and diagnostics fields using functional nanoparticles manufactured in synthesis or biotechnologies is likely to produce many novel insights, which could also lead to the development of therapeutics by controlling cell behaviors.

## Figures and Tables

**Table 1:** Types and characteristics of extracellular vesicles.

Type of EVs	Origin	Size	Surface Markers	Constituents	Function
Exosomes	Endocytosis pathway	40–120	Alix, Tsg101, CD81, CD63	Lipids, nucleic acids, proteins	Cell-cell signaling
Microvesicles	Budding	50–1000	Integrins, selectins, CD40	Lipids, nucleic acids, proteins	Cell-cell signaling
Apoptotic bodies	Cytoplasmic membrane	500–2000	Annexin V, phosphatidylserine	Organelles, nuclear fragments	Phagocytosis induction

Sizes are in nanometers.
